# Impact of Month of Birth on the Risk of Development of Autoimmune Addison's Disease

**DOI:** 10.1210/jc.2016-2392

**Published:** 2016-08-30

**Authors:** Agnieszka Pazderska, Marta Fichna, Anna L. Mitchell, Catherine M. Napier, Earn Gan, Marek Ruchała, Mauro Santibanez-Koref, Simon H. Pearce

**Affiliations:** Institute of Genetic Medicine (A.P., A.L.M., C.M.N., E.G., M..S-K., S.H.P.), Newcastle University, Newcastle upon Tyne, NE1 3BZ United Kingdom; Institute of Human Genetics (M.F.), Polish Academy of Sciences, 60-479, Poznan, Poland; and Department of Endocrinology, Metabolism and Internal Medicine (M.F., M.R.), Poznan University of Medical Sciences, 60-355 Poznan, Poland

## Abstract

**Context:**

The pathogenesis of autoimmune Addison's disease (AAD) is thought to be due to interplay of genetic, immune, and environmental factors. A month-of-birth effect, with increased risk for those born in autumn/winter months, has been described in autoimmune conditions such as type 1 diabetes and autoimmune thyroid disease.

**Objective:**

Month-of-birth effect was investigated in 2 independent cohorts of AAD subjects.

**Design, Setting, and Patients:**

The monthly distribution of birth in AAD patients was compared with that of the general population using the cosinor test. A total of 415 AAD subjects from the United Kingdom cohort were compared with 8 180 180 United Kingdom births, and 231 AAD subjects from the Polish cohort were compared with 2 421 384 Polish births.

**Main Outcome Measures:**

Association between month of birth and the susceptibility to AAD.

**Results:**

In the entire cohort of AAD subjects, month-of-birth distribution analysis showed significant periodicity with peak of births in December and trough in May (*P* = .028). Analysis of the odds ratio distribution based on month of birth in 2 cohorts of patients with AAD versus the general population revealed a December peak and May trough, and January peak and July trough, in the United Kingdom and Polish cohorts, respectively.

**Conclusion:**

For the first time, we demonstrate that month of birth exerts an effect on the risk of developing AAD, with excess risk in individuals born in winter months and a protective effect when born in the summer. Exposure to seasonal viral infections in the perinatal period, coupled with vitamin D deficiency, could lead to dysregulation of innate immunity affecting the risk of developing AAD.

## Abstract (precis)

We show for the first time that month of birth exerts an effect on the risk of developing AAD, with excess risk in individuals born in winter months and a protective effect when born in the summer.

Autoimmune Addison's disease (AAD) is a rare disorder with a reported prevalence of 93–140 per million (1). Its pathogenesis remains incompletely understood and is thought to be due to a complex interplay between genetic, immune, and environmental factors (2). Although a number of susceptibility alleles for the disease have been identified to date, data on the environmental factors implicated in the pathophysiology are completely lacking (3–8).

AAD often occurs with other organ-specific autoimmune disorders; among the most prevalent are autoimmune thyroid disease, type 1 diabetes and vitiligo (9). Given the strong association between these disorders, it can be postulated that they share a common trigger for an autoimmune process directed against different tissue-specific autoantigens.

A month-of-birth effect has been described in a number of autoimmune disorders, including type 1 diabetes (10) and autoimmune thyroid disease; however, conflicting data regarding the latter have recently emerged (11, 12).

One of the proposed links between an individual's birth month and an autoimmune diathesis later in life is season-related environmental exposure, or lack thereof, in the perinatal period. According to the fetal origins of adult disease hypothesis, adverse in utero environmental exposure can lead to maladaptive responses which result in alterations in organ- and tissue-specific functions, which may increase the risk of adult disease (13). Among the important environmental factors that exhibit seasonal variability and which may be implicated in the development of autoimmunity are viral infections (14), UVB radiation and vitamin D_3_ exposure (15, 16), maternal nutrition (16), allergen exposure (17), and air pollution (18).

To our knowledge, there are no data relating to a potential month-of-birth effect in AAD. We sought to ascertain whether such an association existed and, if so, whether a seasonal predilection for the development of AAD could provide the basis for an exploration of potential environmental underpinning for the condition.

## Subjects and Methods

Month-of-birth effect was analyzed in United Kingdom and Polish cohorts of patients with AAD.

The diagnosis of AAD was based on typical symptoms and signs, confirmed by either the subject having a low basal cortisol with a high adrenocorticotrophic hormone concentration or a subnormal response to the short synacthen test (250 mcg parenteral synthetic adrenocorticotrophic hormone_1–24_). In most patients, autoimmune etiology was confirmed by the detection of 21-hydroxylase antibodies, or antiadrenal antibodies in cases with more longstanding diagnosis. Patients with primary adrenal failure due to adrenal gland infiltration or infection, bilateral adrenalectomies, congenital adrenal hyperplasia, or with secondary adrenal failure were excluded from this study.

The data for the United Kingdom cohort were extracted from a local database at the Institute of Genetic Medicine, Newcastle University. The United Kingdom cohort comprised 415 patients with AAD (312 females and 103 males). This cohort included patients who were recruited through local endocrinology clinics in the North East region and patients from other regions of United Kingdom who responded to an invitation, via the Addison's Disease Self-Help Group, to participate in research at the Institute of Genetic Medicine in Newcastle. The data were compared with the pattern of total live births for England and Wales during 1963–1972 (n = 8 180 180). Demographic information was obtained from the Office for National Statistics.

The data for the Polish cohort were extracted from the patients' records at the Department of Endocrinology, Metabolism and Internal Medicine, Poznan University of Medical Sciences. The Polish cohort comprised 231 AAD patients (167 females and 64 males) attending the University Hospital and other endocrine clinics in the Wielkopolska region. The control data regarding total live births in Poland were derived from the Polish Statistical Annals for the years 1980, 1990, 2000, 2010, and 2012 (n = 2 421 384).

Data regarding year of birth, year of AAD diagnosis, and the age at diagnosis are presented in Table 1; 46% and 27% of subjects had isolated AAD in the United Kingdom and Polish cohorts, respectively. The reminder had at least one other autoimmune condition. Data regarding these conditions are presented in Table 2, these data were not available for 5 patients from the polish cohort.

**Table 1.** Patient Characteristics

AAD Cohort	Gender (F/M)	Year of Birth^a^	Year of Diagnosis^a^	Age at Diagnosis^a^
United Kingdom	312/103	1953 (1914–2002)	1996 (1948–2015)	38 (10–83)
Polish	167/64	1967 (1924–1996)	1999 (1954–2014)	36 (9–81)

a Data expressed as median with ranges.

**Table 2.** Concomitant Autoimmune Conditions in AAD Subjects

AAD Patents	United Kingdom Cohort Number (%)	Polish Cohort Number (%)
Isolated AAD	192 (46)	60 (27)
Autoimmune thyroid disease	167 (40)	156 (69.6)
Hashimoto thyroiditis	132 (32)	123 (54.9)
Graves' disease	35 (8.4)	33 (14.7)
Chronic atrophic gastritis ± pernicious anaemia	18 (4.4)	27 (12.1)
Type 1 diabetes	25 (6)	22 (9.8)
Celiac disease	8 (1.9)	3 (1.3)
Hypergonadotrophic hypogonadism	42 (10)	15 (6.7)
Vitiligo	15 (3.6)	14 (6.3)
Alopecia	No data	4 (1.8)
Rheumatoid arthritis	8 (1.9)	No data

This study was carried out with approval of the Leeds East Ethics Committee (reference number 05/Q1206/144) and the ethical committee at Poznan University of Medical Sciences (decision 457/14).

Statistical analysis was conducted using SPSS Version 22 and R (19) using the package season. Month-by-month variation was screened for using X^2^, with odds ratios (ORs) and 95% confidence intervals (CIs) calculated, to compare the birth rates for each month in subjects with AAD and the control population. This analysis was performed separately for the United Kingdom and polish cohorts. Logistic regression fitting a cosinor model was used to estimate the annual seasonal trend in the month-of-birth distribution across all 12 months (20, 21).

## Results

To assess whether month of birth influences susceptibility to AAD, we compared the distribution of month of birth in all patients with AAD with that of the control population. Using the cosinor test, a significant signal for periodicity was observed after adjustment for gender and country of origin (*P* = .028). When the cohorts were analyzed separately, this signal retained its significance in the Polish cohort only (*P* = .042). AAD patients showed a peak of births in December and nadir in June.

**Figure 1.**
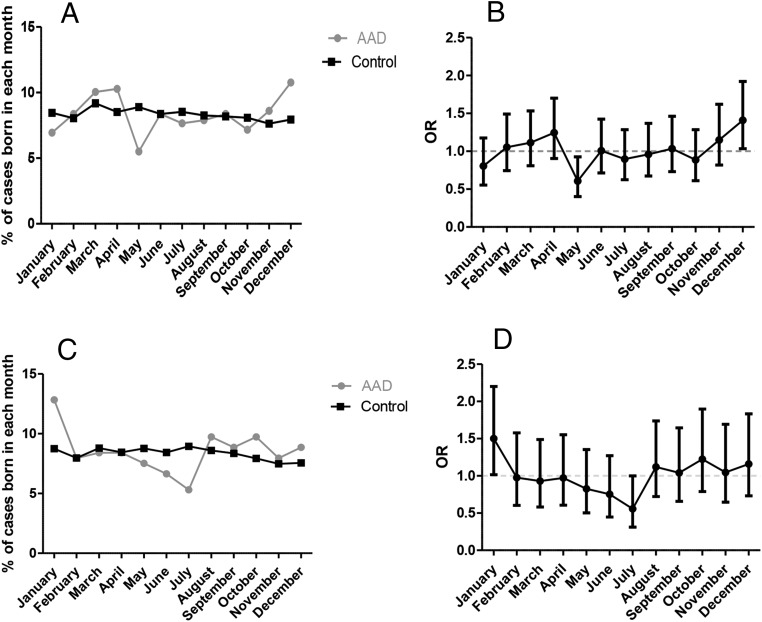
A, Month-of-birth distribution for the United Kingdom cohort. B, Odds ratio (OR) distribution with 95% confidence intervals (CI) based on month of birth in United Kingdom AAD patients vs general population. A significant peak in December (*P* = .029) and a trough in May (*P* = .019) can be observed. C, Month-of-birth distribution for the Polish cohort. D, OR distribution with 95% CI based on month of birth in Polish AAD patients vs general population. A significant peak in January (*P* = .04) and a trough in July (*P* = .046) can be observed.

When monthly ORs were calculated, an increased birth rate in December (*P* = .029, OR 1.41, 95% CI 1.035–1.92) and reduced birth rate in May (*P* = .019, OR 0.61, 95% CI 0.4–0.93) was detected in the United Kingdom AAD cohort (Figure 1B) compared with the general United Kingdom population. In a gender-stratified analysis, a statistically significant peak was found in December only in the United Kingdom female AAD subjects (*P* = .032, OR 1.47, 95% CI 1.03–2.01).

In the Polish AAD cohort, an increased birth rate in January (*P* = .04, OR 1.5, 95% CI 1.02–2.1) and decreased birth rate in July (*P* = .046, OR 0.56, 95% CI 0.31–0.99) was detected (Figure 1D) compared with the general polish population. In a gender-stratified analysis, a statistically significant peak was found in January only in the Polish female AAD subjects (*P* = .009, OR 1.8, 95% CI 1.15–2.73).

In order to assess the possible effect of latitude on the seasonality of birth in AAD, we divided the entire cohort into 2 groups according to latitude. A latitude of 52° North was chosen, because it provided a reasonable bisection of both cohorts. The highest and the lowest monthly ORs were found north of 52° latitude; however, 95% CIs were overlapping, indicating no significant difference (Supplemental Figure 1).

## Discussion

To our knowledge, this is the first report of a month-of-birth effect on the risk of developing autoimmune AAD. Our study showed similar trends in seasonality patterns for 2 independent cohorts of patients with AAD. There were a smaller number of AAD subjects born in summer months, with a nadir in May for the United Kingdom and July for Polish subjects, and a greater number of AAD subjects born in winter months, with a peak in December in the United Kingdom, and January in the Polish cohort. Although in the United Kingdom AAD cohort there is some heterogeneity in monthly ORs, this is not statistically significant and likely represents a stochastic effect in a dataset that becomes smaller when split in 12 ways. When looking at fluctuations across all 12 months in the entire cohort of patients, a similar pattern with a trough in summer and peak in winter was observed. This is the first study of this kind and as such is exploratory in nature and warrants further interrogation in other cohorts of AAD subjects.

The month-of-birth effect in immune mediated diseases has been long explored and a number of such studies show a reverse pattern of seasonality with a peak in spring/summer and a trough in autumn/winter (15, 22). However, a number of studies on autoimmune conditions commonly associated with AAD reported similar patterns to that observed in our study, with a peak in the number of births observed in autumn and winter in populations of patients with Graves' hyperthyroidism and Hashimoto's hypothyroidism (11, 12). It is plausible that in different autoimmune diseases various environmental triggers play a role during different stages of gestational and early postnatal development to influence disease susceptibility later in life.

Our findings mirror the observation that the peak in type 1 diabetes incidence occurs in autumn and winter months, which has been linked to the increase in viral infections observed during this period of year (23). A number of viral infections such as norovirus, rotavirus, influenza and respiratory syncytial virus exhibit marked wintertime seasonality (24). Innate immunity is concerned with the defense of the host from infection and has been implicated in the pathophysiology of AAD. A number of susceptibility loci for AAD in the innate immune pathways have been discovered including *NLRP1*, *CLEC16A*, *VDR*, and *CYP27B1* (4, 25–27). In addition, experimental data also support the role of innate immunity in the pathophysiology of AAD (28). It is plausible that early priming of immune cells and immune mechanisms induced by exposure to various (seasonally influenced) pathogens can influence disease risk in individuals who carry multiple other susceptibility alleles. Although most patients with AAD are diagnosed between the third and fifth decades of life, this disorder is known to have prolonged subclinical phase lasting for many years (29, 30). This raises the possibility that a similar, seasonally influenced infective trigger may be implicated in its pathogenesis but with a longer clinical latency than type 1 diabetes.

One of the postulated mechanisms explaining a month-of-birth effect in autoimmune disease is the role that vitamin D deficiency might play in the pathophysiology of such diseases (31). Several reports have demonstrated that 1,25-dihydroxyvitamin D signaling regulates both innate and adaptive immunity in humans (32). This hypothesis is particularly plausible in the case of AAD because of the known association of the disease with polymorphisms in both the vitamin D receptor (*VDR*) gene and the *CYP27B1* gene encoding 1-α-hydroxylase converting 25-hydroxyvitamin D into its active form 1,25-dihydroxyvitamin D (8, 27, 33). To explore the possible effect of UVB radiation exposure, which is known to influence vitamin D status, on the seasonality pattern observed in AAD, we compared monthly ORs (Supplemental Figure 1) dividing the cohort into 2 groups according to geographical latitude. Although the effect of seasonality seemed to be more pronounced above 52° North, this did not reach statistical significance. Further studies are required to investigate the association of perinatal vitamin D status and the risk for AAD. Verifying vitamin D deficiency and AAD risk by ascertaining 25 (OH)D status in infancy, however, will be difficult given the rarity of the condition and the fact that diagnosis is usually made in individuals in their third to fifth decade.

We acknowledge there are potential limitations to our study. Firstly, given the inherent rarity of this condition, our sample size is relatively small. This could potentially limit the ability to detect differences, particularly in relation to the subgroup analysis. Nonetheless, ours represents one of the largest cohorts of this condition published to date. Secondly, the subjects in our study were not recruited from a formal national or regional registry, which could potentially lead to bias, although we feel that this is unlikely and that the described trend in seasonality is a true finding, given the similar pattern in 2 independent cohorts of patients recruited in 2 different countries. Further studies in large cohorts of AAD patients looking at an association of month of birth and the risk of development of the disease would be useful.

Lastly, our controls obtained from the Office for National Statistics and the Polish Statistical Annals inevitably include subjects with AAD. However, given the rarity of the disease, the expected number of AAD cases in the control population would be very small and would not influence the overall result.

In conclusion, our study demonstrates that month of birth exerts an effect on the risk of developing AAD, with excess risk in individuals born in winter months and a protective effect when born in the summer. This is the first such environmental influence documented in the pathogenesis of AAD. Exposure to seasonal viral infections in the perinatal period coupled with vitamin D deficiency leading to dysregulation of innate immunity could be the potential initial triggers for the eventual development of AAD, although this requires further exploration.
